# Ultrasound Assessment of Adnexal Pathology: Standardized Methods and Different Levels of Experience

**DOI:** 10.3390/medicina57070708

**Published:** 2021-07-13

**Authors:** Indrė Tavoraitė, Laura Kronlachner, Gina Opolskienė, Daiva Bartkevičienė

**Affiliations:** 1 Faculty of Medicine, Vilnius University, 03101 Vilnius, Lithuania; indre.tavoraite@gmail.com; 2 Centre of Obstetrics and Gynecology, Faculty of Medicine, Institute of Clinical Medicine, Vilnius University, 03101 Vilnius, Lithuania; laura@endobiogenika.lt (L.K.); gina.opolskiene@gmail.com (G.O.)

**Keywords:** adnexal pathology, standardized methods, ultrasound, experience

## Abstract

*Background and objectives*: An expert’s subjective assessment is still the most reliable evaluation of adnexal pathology, thus raising the need for methods less dependent on the examiner’s experience. The aim of this study was to evaluate the performance of standardized methods when applied by examiners with different levels of experience and to suggest the most suitable method for less-experienced gynecologists. *Materials and methods*: This single-center retrospective study included 50 cases of histologically proven first-time benign or malignant adnexal pathology. Three examiners evaluated the same transvaginal ultrasound images: an expert (level III), a 4th year resident in gynecology (level I), and a final year medical student after basic training (labeled as level 0). The assessment methods included subjective evaluation, Simple Rules (SR) with and without algorithm, ADNEX and Gynecologic Imaging Reporting and Data System (GI-RADS) models. Sensitivity, specificity, accuracy, positive and negative predictive values with 95% confidence interval were calculated. *Results*: Out of 50 cases, 33 (66%) were benign and 17 (34%) were malignant adnexal masses. Using only SR, level III could classify 48 (96%), level I—41 (82%) and level 0—40 (80%) adnexal lesions. Using SR and algorithm, the performance improved the most for all levels and yielded sensitivity and specificity of 100% for level III, 100% and 97% for level I, 94.4% and 100% for level 0, respectively. Compared to subjective assessment, ADNEX lowered the accuracy of level III evaluation from 97.9% to 88% and GI-RADS had no impact. ADNEX and GI-RADS improved the sensitivity up to 100% for the less experienced; however, the specificity and accuracy were notably decreased. *Conclusions*: SR and SR+ algorithm have the most potential to improve not only sensitivity, but also specificity and accuracy, irrespective of the experience level. ADNEX and GI-RADS can yield sensitivity of 100%; however, the accuracy is decreased.

## 1. Introduction

Adnexal pathology accounts for numerous gynecologic problems, varying from endometriomas, hydrosalpinges and teratomas to malignant tumors. In everyday practice the first-line visual modality for gynecologic examination is ultrasound and the most extensive research on this topic has been conducted by The International Ovarian Tumour Analysis (IOTA) group founded in 1999. Since then, the diagnostic ultrasound approach has been considerably reformed from subjective experience-based evaluation into more structural evidence-based algorithms, such as Simple Rules (SR), the ADNEX, LR1, or LR2 risk models. Additionally, The Gynecologic Imaging-Reporting and Data System (GI-RADS) was introduced more specifically for the evaluation by radiologists. Nevertheless, today a subjective assessment by an experienced examiner is still the most reliable method of evaluation. As determination of a lesion being either benign or malignant is one of the first and the most important steps in management, there is a need for evaluation techniques that would be the least dependent on examiner’s experience and yield the least false negative results.

Most of the articles available in English within the last 5 years describe the performance of IOTA, ADNEX, and GI-RADS models based only on experts’ also known as level III [1] examiners’ evaluation data. Only several [2,3,4,5,6,7] conduct research including less experienced trainees or residents, yet only describing the performance of SR with or without subjective evaluation. No articles were found describing GI-RADS or ADNEX performance when applied by level I or even less experienced examiners.

The main purpose of this study was to evaluate the performance in diagnostic precision of subjective assessment, IOTA Simple rules, ADNEX and GI-RADS models when applied by examiners of varying expertise levels: an expert gynecologist with over 20 years of experience and completed IOTA exam, a 4th year resident of gynecology and a final year medical student. Furthermore, we aimed to determine the most suitable evaluation method for less experienced gynecologists. To the extent of our knowledge, this is the first study available in English to include examiners of such different levels of experience combined with analysis of four different methods of adnexal pathology assessment.

## 2. Materials and Methods

This is a single-center retrospective study conducted at Vilnius University Hospital Santaros Klinikos (VUL SK) which is a tertiary oncology center.

### 2.1. Patient and Data Collection

Out of 457 patients hospitalized due to adnexal mass pathology in VUL SK from January of 2019 to March of 2020, only 50 were eligible for inclusion. Criteria for inclusion: first time diagnosed adnexal pathology, good quality ultrasound images saved in the database, images were obtained by the same specialist and the same ultrasound machine, available histology results after surgery.

### 2.2. Ultrasound Examination

The ultrasound examination was performed by an experienced gynecologist using a transvaginal probe of the Voluson E8 ultrasound machine. The intensity or absence of blood flow in the lesion was evaluated. All images were saved in the database of VUL SK.

### 2.3. Evaluation of Ultrasound Images

Three examiners evaluated the same ultrasound images. All examiners were blind to the histology results and were only aware of the factors necessary for the assessment models, such as age and CA125 concentration (if available and only when applying the ADNEX model). The first examiner was an expert with over 20 years of experience who completed the IOTA examination (level III [1]), the second examiner was a 4th year resident in gynecology (level I) and the third examiner was a final year medical student with no experience in gynecological ultrasound evaluation (we labeled it as level 0). The second and third examiners prepared for the evaluation by watching a lecture “Terminology and using Simple Ultrasound Based rules to classify Ovarian Pathology“ by prof. Timmerman as well as studying articles about terminology, use of SR, and the ADNEX and GI-RADS models [8,9,10,11,12,13].

First, an examiner did a subjective evaluation of the lesion: whether it is benign or malignant (including borderline) as well as which specific pathology is suspected.

Second, using GI-RADS criteria (Table 1) [14] an examiner determined which of the 5 categories the lesion can be assigned to. Findings suggesting malignancy are thick papillary projections, thick septations, solid areas, central vascularization, and ascites.

Third, the SR method was applied [15]. The SR method includes five M criteria describing a malignant tumor (M1—irregular solid tumor; M2—presence of ascites; M3—at least four papillary structures; M4—irregular multilocular solid tumor with largest diameter ≥ 100 mm; M5—very strong blood flow, color score 4) and five B criteria describing a benign tumor (B1—unilocular; B2—presence of solid components where the largest solid component has a largest diameter < 7 mm; B3—presence of acoustic shadows; B4—smooth multilocular tumor with largest diameter < 100 mm; B5—no blood flow, color score 1). If the lesion has at least one M criterion and no B criteria, it is classified as malignant. If the lesion has at least one B criterion and no M criteria, it is classified as benign. If neither B nor M features or both B and M features can be applied, the lesion cannot be classified [15]. As there were some inconclusive cases when applying SR, only those with a conclusive diagnosis were included in calculations assessing SR. Therefore, those results are not suitable for comparison with subjective evaluation, ADNEX, or GI-RADS results.

In order to classify all the cases enrolled, the following algorithm was used (SR + algorithm). In cases of undetermined lesions when level 0 and I examiners were applying SR, an expert’s SR evaluation was used as final diagnosis. If the expert’s evaluation using SR was also inconclusive, the lesion was classified based on the expert’s subjective evaluation. The diagnostic indices were also calculated for the SR + algorithm. Thus, these indices can be directly compared with those of the other methods of evaluation.

Finally, the ADNEX model using a calculator from the official IOTA website was applied [16]. The calculator requires the following information: age, oncology center (yes/no), maximal diameter of the lesion (mm), maximal diameter of the largest solid part (mm), more than 10 locules (yes/no), number of papillations (none/one/two/three/more than three), acoustic shadows present (yes/no), ascites present (yes/no), serum CA-125 (if available). For some patients serum CA-125 concentration data was not available; however, studies show that inclusion of CA-125 does not alter the discrimination between benign and malignant adnexal masses [17]. The given results provide the probability of the lesion being in each of the following categories: benign; malignant; each type of malignancy (borderline, stage I, stage II-IV, metastatic). The highest given percentage showed which category the lesion is in. The cut off value for defining malignancy was a risk of 10% or above.

If the patient had more than one adnexal mass, the bigger or more complex lesion was evaluated. The borderline tumors were assigned to the group of malignant adnexal masses.

### 2.4. Histopathology

Histology results were the reference standard for the diagnosis of all adnexal masses. The histopathologic analysis of adnexal tissue was conducted in the National Center of Pathology, Affiliate of VUL SK.

### 2.5. Statistical Analysis

The diagnostic performance of the subjective evaluation, SR, ADNEX, and GI-RADS was presented as sensitivity, specificity, accuracy, and positive and negative predictive values (PPV, NPV) with 95% confidence interval. The Microsoft Office Excel and MedCalc [18] were used for calculations.

## 3. Results

The mean age of the patients was 46.7 (standard deviation, SD = 15.6), ranging from 25 to 83. Out of 50 patients enrolled in the study, 33 (66%) had benign and 17 (34%) had malignant histologically confirmed adnexal masses. The majority of benign lesions consisted of endometriomas (8), teratomas (6), and serous cystadenomas (5). Others included corpus luteum (2) or follicular cysts (2), mucous (2) or rete ovarii (3) cystadenomas, mucous adenofibroma (1), fibroma (1), hydrosalpinx (2), and paratubal cyst (1). The majority of malignant lesions were serous carcinoma (6) and granular cell carcinoma (4). Others included serous papillary carcinoma (1), non-differentiated carcinoma (1), serous borderline tumors (3), endometrioid borderline tumor (1), and metastatic tumor of lung adenocarcinoma (1).

**Figure 1:**
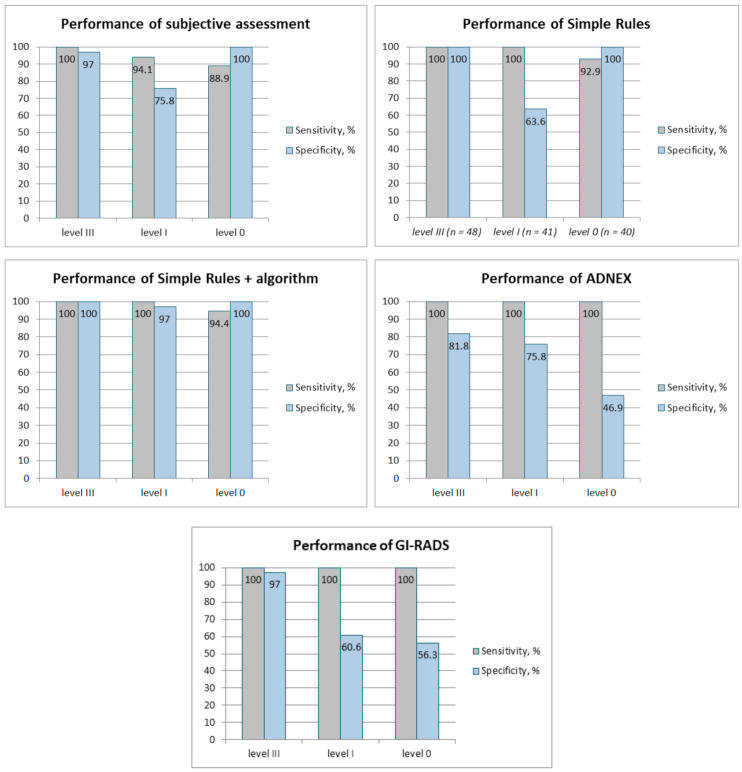
Sensitivity and specificity of different methods based on levels of experience.

The diagnostic performance of ultrasound evaluation models is presented in Table 2, Table 3 and Table 4 and Figure 1.

### 3.1. Subjective Assessment

Sensitivity of subjective assessment improved as the level of experience increased (level 0: 88.9%; level I: 94.1%; level III: 100%); however, the specificity was varying (level 0: 100%, level I: 75.8%, level III: 97%). The level 0 examiner was more likely to have false negative results of subjective evaluation. The subjective evaluation of level III showed very high sensitivity and specificity, 100% and 97% respectively.

Based on accuracy, the best diagnostic performance was of level III evaluation, as expected. However, level 0 examination showed higher accuracy (96%) compared to level I (82%). The reason for this might be that the level I evaluator, who was in residency training, was aiming for high sensitivity rather than high specificity, expecting the least false negative results.

### 3.2. Simple Rules

During level III examiner’s evaluation, the SR were conclusive in 48 (96%) cases with 2 (4%) cases left uncategorized. However, level I and 0 examiners could achieve conclusive diagnoses using SR in only 41 (82%) and 40 (80%) cases, respectively, resulting in approximately 1 out of 5 patients being referred to the expert.

By using the SR + algorithm, described in the Section 1, the expert classification resulted in 100% accuracy. Level I examiner achieved 100% sensitivity with no false negative results when using SR + algorithm. Although level 0 evaluation resulted in some false negative diagnoses (5.6%) when applying the SR + algorithm, the sensitivity (94.4%) and specificity (100%) are very high for an examiner with almost no experience and shows objectivity of the method.

### 3.3. Adnex Model

Using the ADNEX model with a cut-off value of 10% all the examiners achieved 100% sensitivity, resulting in no false negative results. However, level 0 evaluation showed the least specificity (46.9%), accuracy (66%), and PPV (51.4%) meaning high numbers of false positive results. In comparison, level I and III resulted in higher specificity, 75.8% and 81.8%, and accuracy, 84% and 88%, respectively. However, when compared to the subjective evaluation, the experts’ results significantly decreased in specificity.

### 3.4. GI-RADS Model

Results of the GI-RADS model show similarities with the ADNEX model due to achieved sensitivity of 100% by all examiners. Level III evaluation showed high specificity (97%) and accuracy (97.9%), identical to that of subjective assessment. However, level I evaluation resulted in much lower specificity (60.6%) and accuracy (74%) compared to ADNEX, similar to that of level 0 (56.3% and 72%, respectively). Level 0 and I showed lower accuracy rates compared to subjective assessment.

## 4. Discussion

The subjective assessment of an expert gynecologist is still the most sensitive method of adnexal mass evaluation. Our results are conclusive with the literature, showing 100% sensitivity of expert’s examination resulting in no false negative results, irrespective of the method. Opposingly, for the less experienced gynecologists a standardized method for evaluation might notably elevate sensitivity and other indices of performance. Hence, the aim of our study was to assess how successful various ultrasound evaluation methods are for less experienced examiners in comparison to the expert.

Sensitivity and NPV are the most important indicators of the methods’ performance for the less experienced gynecologists as they accurately represent rates of false negatives. All methods tested in this study improved sensitivity and NPV for the less experienced compared to subjective assessment.

There is a lack of studies focused on GI-RADS and ADNEX performance when applied by inexperienced gynecologists. Although these methods achieved 100% sensitivity for less experienced examiners in our study, the specificity and accuracy were notably lower in contrast to subjective assessment. Based on the experts’ evaluation data, the sensitivity, specificity, and accuracy of GI-RADS vary in the literature: 92.7–96.4%; 84.3–97.5%; 89.3–95.7%, respectively [19,20,21], and our results for level III examiner are similar.

The main issue when applying GI-RADS during our study was the lack of clarification on malignancy criteria application which led to a significant loss of specificity and more false positive results for less experienced examiners. No accurate instructions could be found in literature about applying malignancy criteria when assigning lesions to category IV. For instance, even though teratomas were recognized and could be categorized as category III, less experienced examiners followed the instructions and interpreted the solid areas as a criteria for malignancy, thus having to classify such lesions as category IV. Additionally, this method of evaluation still requires more experience compared to others. In order to assign lesions to categories II or III, the evaluator is required to know the typical ultrasound signs and differentiate masses of functional origin or benign ovarian lesions.

Similarly to the GI-RADS, ADNEX model resulted in markedly decreased specificity when compared to subjective evaluation, even for the expert. The original study of Van Calster et al. based on experts’ evaluations reported 96.5% sensitivity and 71.3% specificity [22]. According to Jeong et al. the diagnostic accuracy of the ADNEX when applied by the expert does not differ significantly from the expert’s subjective evaluation and can achieve 90% sensitivity and 81.6% specificity [23]. Other studies report sensitivity and specificity of experienced gynecologists using an ADNEX model (10% cut-off) ranging 88.6–97.3% and 55.5–94.4%, respectively. Our results show 100% sensitivity for all examiners; however, specificity and thus accuracy are notably reduced, especially for inexperienced gynecologists (specificity of level 0: 46.9%; level I: 75.8%). The pattern of such varying specificity might be the result of several ADNEX criteria which can be measured differently by examiners even for the same patient. We suggest that the ADNEX model might be the most beneficial for the less experienced when malignancy differentiation has to be made quickly without referring to the expert as well as in undetermined cases after SR while having in mind the increased risk for more false positive results. This method is also suitable for oncology centers when differentiation between cancer stages is required for planning the treatment.

Based on our findings, SR has the most potential to be the first choice method for the less experienced. Furthermore, SR and SR + algorithm were the only methods improving expert’s specificity, PPV and accuracy. The main problem arising with SR use is the amount of inconclusive results. Ning et al. reported that an inexperienced gynecologist using SR yielded conclusive results in 79.4% of the cases while the expert had 92% conclusive results [7], which is similar to our data. This means high numbers of cases being referred to the experts and reduced overall work efficiency. Nevertheless, the valuable SR improvement of diagnostic performance when applied by the less experienced in comparison to subjective assessment has been reported. Results of Ning et al. study [7] show increase in sensitivity when SR were applied for both inexperienced gynecologists and experts, from 72.4% to 96.7% and from 96.2% to 100%, respectively. However, they reported a slight reduction in specificity for both types of gynecologists, from 88.8% to 87.3% for the inexperienced and from 96.3% to 94% for the expert. In contrast, our study showed that applying SR and SR + algorithm improved both sensitivity and specificity as well as overall diagnostic performance for all examiners when compared to subjective assessment with the exception for level I examiner when specificity was improved only by using SR + algorithm. Similarly, Knafel et al. [2] reported the value of SR + algorithm for the level I examiner’s evaluation, when the diagnosis for the inconclusive cases after SR was defined based on the expert’s subjective evaluation. The sensitivity and specificity of SR + algorithm in comparison to SR alone increased from 95.4 to 96.3 and from 94.4 to 95.1, respectively. However, in this study they reported much greater SR specificity for the level I examiner.

The limitations of our study are the following: a single-center study, small number of patients included, only one examiner of each level of experience, lack of level II examiner, and the ultrasound was not performed personally by each examiner. Thus, an examiner could not visualize the mass in different planes and scan thoroughly. Some improvements could be made for future research such as conducting a prospective multi-center analysis including more sonographers and testing evaluation methods in real time by performing ultrasound personally.

## 5. Conclusions

All methods were effective in achieving high sensitivity when used by less experienced examiners, which is crucial for limiting false negative results. Therefore, less experienced examiners could choose any method for improving sensitivity and NPV. For the expert, all methods including subjective evaluation resulted in 100% sensitivity; however, only SR and SR + algorithm improved specificity and accuracy. More studies should be conducted focusing on less experienced gynecologists, especially for the GI-RADS and ADNEX models. The latter methods have a limited ability to improve diagnostic performance for the inexperienced. However, SR and SR + algorithm showed the highest potential for improving all of the performance indices, including sensitivity, specificity and overall accuracy, for a gynecologist regardless of his experience.

## Figures and Tables

**Table 1:** The Gynecologic Imaging-Reporting and Data System (GI-RADS) classification.

Classification	Risk of Malignancy	Morphology
GI-RADS 1	Definitively benign	Normal ovaries, no adnexal masses identified
GI-RADS 2	Very probably benign	Adnexal masses of functional origin, such as follicles, hemorrhagic cysts.
GI-RADS 3	Probably benign	Adnexal lesions thought to be benign, such as endometrioma, hydrosalpinx, teratoma, simple or paraovarian cyst, or finding of pelvic inflammatory disease.
GI-RADS 4	Probably malignant	Adnexal masses not included in the above groups and with 1 or 2 malignancy suggesting features.
GI-RADS 5	Very probably malignant	Adnexal masses with 3 or more malignancy suggesting features.

**Table 2:** Diagnostic performance of models based on expert’s (level III) evaluation.

Level III Examiner	Sensitivity % (95% CI)	Specificity % (95% CI)	Accuracy % (95% CI)	Positive Predictive Value % (95% CI)	Negative Predictive Value % (95% CI)
Subjective evaluation	100 (78.2–100)	97 (84.2–99.9)	97.9 (88.9–100)	93.8 (68.5–99)	100
GI-RADS	100 (78.2–100)	97 (84.2–99.9)	97.9 (88.9–100)	93.8 (68.5–99)	100
SR (*n* = 48)	100 (78.2–100)	100 (89.4–100)	100 (92.9–100)	100	100
SR + algorithm	100 (80.5–100)	100 (89.4–100)	100 (92.6–100)	100	100
ADNEX (cut off 10%)	100 (80.5–100)	81.8 (64.5–93)	88 (76–95)	73.9 (57.9–85.4)	100

**Table 3:** Diagnostic performance of models based on resident’s (level I) evaluation.

Level I Examiner	Sensitivity % (95% CI)	Specificity % (95% CI)	Accuracy % (95% CI)	Positive Predictive Value % (95% CI)	Negative Predictive Value % (95% CI)
Subjective evaluation	94.1 (71.3–99.9)	75.8 (57.7–88.9)	82 (68.6–91.4)	66.7 (52–78.7)	96.2 (78.7–99.4)
GI-RADS	100 (80.5–100)	60.6 (42.1–77.1)	74 (59.7–85.4)	56.7 (46.1–66.6)	100
SR (*n* = 41)	100 (78.2–100)	63.6 (45.1–79.6)	76 (61.8–87)	58.6 (47.4–69)	100
SR + algorithm	100 (80.5–100)	97 (84.2–99.9)	98 (89.4–100)	94.4 (71.1–99.2)	100
ADNEX (cut off 10%)	100 (80.5–100)	75.8 (57.7–88.9)	84 (70.9–92.8)	68 (53.8–79.5)	100

**Table 4:** Diagnostic performance of models based on student’s (level 0) evaluation.

Level 0 Examiner	Sensitivity% (95% CI)	Specificity% (95% CI)	Accuracy% (95% CI)	Positive Predictive Value % (95% CI)	Negative Predictive Value % (95% CI)
Subjective evaluation	88.9 (65.3–98.6)	100 (89.1–100)	96 (86.3–99.5)	100	94.1 (81.2–98.3)
GI-RADS	100 (81.5–100)	56.3 (37.7–73.6)	72 (57.5–83.8)	56.3 (46.5–65.6)	100
SR (*n* = 40)	92.9 (66.1–99.8)	100 (86.8–100)	97.5 (86.8–99.9)	100	96.3 (79.7–99.4)
SR + algorithm	94.4 (72.7–99.9)	100 (89.1–100)	98 (89.4–100)	100	97 (82.7–99.5)
ADNEX (cut off 10%)	100 (81.5–100)	46.9 (29.1–65.3)	66 (51.2–78.8)	51.4 (43.3–59.5)	100

## Data Availability

The data presented in this study are available on request from the corresponding author. The data are not publicly available and are not uploaded to any repository.
